# Advances in the Analysis of Properties Behaviour of Cement-Based Grouts with High Substitution of Cement with Blast Furnace Slags

**DOI:** 10.3390/ma13030561

**Published:** 2020-01-24

**Authors:** Francisca Perez-Garcia, Maria Dolores Rubio-Cintas, Maria Eugenia Parron-Rubio, Jose Manuel Garcia-Manrique

**Affiliations:** 1 Departamento de Ingeniería Civil, Materiales y Fabricación, Universidad de Málaga, 29071 Málaga, Spain; perez@uma.es (F.P.-G.); josegmo@uma.es (J.M.G.-M.); 2 Departamento de Ingeniería Industrial y Civil, Universidad de Cádiz, 11202 Algeciras, Spain; mariaeugenia.parron@uca.es

**Keywords:** cementitious grout, cement substitution, slag GGBS, waste valorization

## Abstract

This article presents a study of the main properties (consistency, workability, leaching, unsoundness, and mechanical properties) of cement grouts prepared with cement replacement by blast furnace slag (GGBS). Mixtures have been analyzed in the absence of additives and reached high replacement percentages. As shown in the different tests presented, the observed evolution of the resistance and workability of the mixtures makes them very interesting for its application. Different types of cement (CEM-I 42.5 and CEM-I 52.5 R) and different water/binder values (1 and 0.67) are used. The results present opportunities for the steel industry by the intensive valorization of slag waste. The reduction of the use of cement in construction is also one of the key aims of this line of research. Results show improvements in the mechanical response with good fresh state properties for substitution percentages up to 70%. It is verified with leaching analysis that these products have less impact on the environment.

## 1. Introduction

Currently, the overall economic growth has been accompanied by a significant increase in the demand for steel. This entails a considerable increase of by-products and industrial waste generated in its manufacture such as slag. This generation reaches 2.4 million tons per year. Therefore, the accumulation of slag is a form of environmental pollution to be taken into account. Only a small fraction is used as a by-product while the rest goes directly to waste [1].

In the civil engineering industry, the use of grouts is worldwide widespread, where the Portland cement (CP) is the main element positioned into cementitious matrix. The production of conventional Portland cement involves important carbon dioxide emissions. It is estimated at around 7% to 9% of worldwide CO_2_ emissions [2,3,4]. Therefore, it is an important source of air pollution.

The use of slag as a substitute for cement is a strategy that addresses both challenges, which include the reduction of cement demand and the valorization of the slag that is currently treated as waste. In particular, we propose working with blast furnace slag (GGBFS).

GGBFS can be applied as a substitution of the CP, which makes the cementitious grout mixtures more sustainable, reduces pollution, and preserves natural resources [5,6]. There exists some research where the slag has already been used for the manufacture of the slurries as a substitute for the CP but just at low levels [7]. However, the use of these materials with a high substitution percentage is feasible and innovative.

The mixtures made have been executed in our laboratories. The treatment of slags and mixtures are manual without the use of any chemical additive unlike in-plant productions of mixed Portland cements (CEM III). The last objective is to improve their use to obtain 100% recyclables materials. It supposes considerable benefits as reducing construction cost, the need for landfill sites, or their disposal in the river system [8].

In a previous paper, results were presented with this methodology applied for concrete specimens [9]. In that work, with 25% of cement substitution, the importance of the origin of the slag used were studied. Once we selected a proper GGBFS slag, the research has continued to higher percentages and the results are being promising. In this paper, we will focus on the study of the manufacture of cementitious grouts with high levels of GGBFS substitution. We reach substitutions of cement of 90%, which is comparable to CEM III/C.

Special consideration also takes into account the leaching of toxic materials, especially when the GGBFS substitution is used in cement-based grouts for injection applications in land improvement works. The analysis of the degree of dissolutions reached by these components is essential to prove its suitability for these subjects [10,11,12]. Therefore, we will present these tests for some mixture studied.

Recent research shows that grouts can be made by replacing cement with other materials, F. Amahjour et al. (2002) [13] or Pastor et al. (2016) [14] add fly ash and silica fume to increase mechanical strength. In 2015, Fatih Celik et al. [15] investigated the mixture of rice husk ash in cement-based grout. Another field of study is the analysis of parameters as the classification of mechanical properties with sand (Lim et al. [16]).

Different studies also show results carried out with substitutions of blast furnace slags in small percentages by always adding additives or chemical substances. In 2017, Weijie Zhang introduced sodium silicate for a rapid adjustment [17]. Reza Azadi et al. (2013) [18] worked with chemical additives to optimize the grout. They used sodium silicate (Na_2_SiO_3_) to increase resistance, sodium carbonate (Na_2_CO_3_) to reduce bleeding, or triethanolamine (TEA) to promote injection. There are some studies where the substitution of cement by slags is used to obtain different properties such as Sowmini Gopinathan (2018). It concluded that the replacement of cement by slags in slurries can reduce bleeding substantially without affecting the workability of the mixtures [19]. Sanderson (2017) showed that the peak heat and the evolution of heat increased with the rise in GGBS content [20]. Alireza Joshagani (2018) developed high-performance non-shrinking grout (HPNSG) using fly ash, slag, and silica fume [21]. Sha (2018) make mixtures to ensure fluidity, penetrability, and less leaching. In other work, it was used with Portland cement, fly ash, bentonite, a superplasticizer, and water glass [22]. Cui (2019), which are obtained grouts with good workability, reduced costs and less impact on the environment [23]. Debaleena Mukherjee (2019) designed mixtures for pavements with cement grouted bituminous macadam GGBM [24]. Zahofeng Li (2019) examined the effect of incorporating gypsum dihydrate (GN), combustion gas desulfurization plaster (FGD), and phosphogypsum (PG) on the workability and mechanical properties of the slurry materials of red slags with different plaster contents [25]. Xiang (2019) incorporated metakaolin [26]. Other studies used GGBS slags as a cement substitute for the design of different concretes [27,28,29].

In this study, we analyzed cement-based grouts made simply with water, ordinary Portland cement, and GGBFS. Cement has been replaced by high percentages of GGBFS until reaching 90% substitution. With no chemical component addition. The main objective of this work is to investigate the influence of the main parameters, as cement type, water/binder, and percentage of substitution in the mechanical and fresh state behaviour of the mixture. Each combination results in consistence, exudations, flow properties, compression, and a flexion test that is analyzed from 2 to 90 days. High water/binder values have been selected because one of the potential applications of these cement mixtures are those related to improve ground conditions as the Jet-grouting.

Section 2 describes the main issues of the mixtures and the programme of the test realized. Section 3 presents results of the fresh state properties and Section 4 analyses the results of strength properties. In Section 5 and Section 6, other workability properties as unsoundness and leaching behaviour are discussed. Lastly, in Section 7, we summarize the fundamental conclusions of the research.

## 2. Materials

As mentioned, all the specimens are cement-based grout made from tap water, Portland cement, and slag from blast furnaces without additives.

Two Portland Cement (CP) has been used: CEM I 42.5 and CEM I 52.5 R (UNE-EN 197-1) [30], with density: 3.13 g/cm^3^. The slag used has been GGBFS from a well-known origin and produced good results in concrete tests with low percentage substitutions [9]. Some of its properties are density 2.95 g/cm^3^ and specific surface area 4500–4700 cm^2^/gr. Table 1 presents chemical properties of cement and GGBFS used.

**Figure 1:**
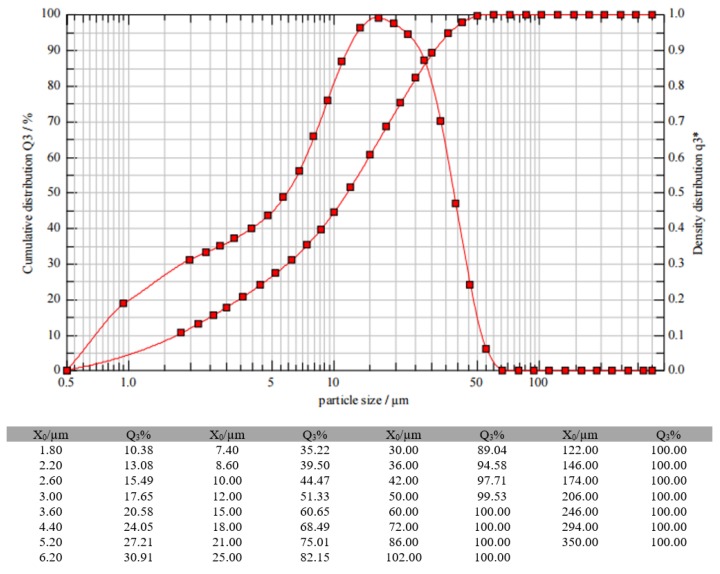
GGBFS particle size analysis.

**Figure 2:**
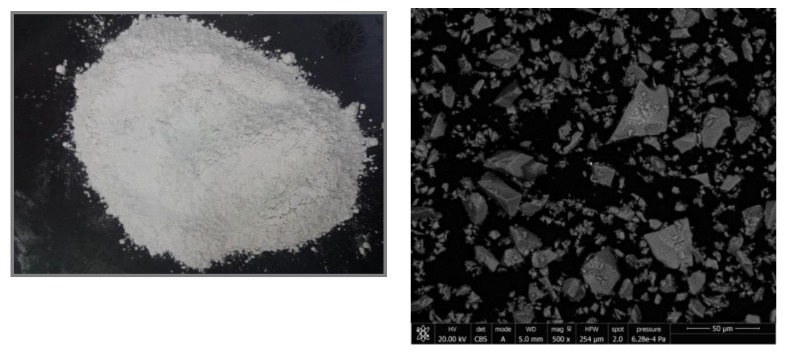
GGBFS SEM micrograph.

In origin, the chosen slag is similar to a sand 0/3 (granulometry from 0 to 3 mm) with high moisture content (8%–10%). In this case, it is subjected to a drying and milling process in the factory itself. The humidity favors the milling process in special vertical roller mills. The particle size analysis of the final slag generated is presented in Figure 1 where μo is the particle size in μm, Q_3_ is the percentage of particles, and q^3^* is the density distribution depending on particle size. A representative electronic micrograph (SEM) of the GGBFS is shown in Figure 2.

The tests have been organized around four different series based on four combinations of cement and water to binder values. Series A includes specimens with CEM-I 42.5 and w/b of 1, Series B includes specimens with CEM-I 52.5 R and w/b of 1. Series C includes specimens with CEM-I 42.5 and w/b of 0.67, and Series D includes specimens with CEM-I 52.5 R and w/b of 0.67. Each series has a reference mixture with no substitution (A0, B0, C0, and D0) and others were the percentage of slag substituted, which is referred to by the identification number. For example, id B90 is a series B specimen with 90% of cement substituted by GGBFS slag. The mixtures were prepared in the laboratory at a temperature of 24 to 26 °C and 40% of relative humidity. All of them were prepared according to the European standard used in the manufacture of cement grouts UNE-447 [31]. An electric mixer of robust construction according to EN 196/1 [32] were used in the manufacture. It takes 90 s with the slag and then 180 s more with the addition of water. More than 300 prismatic samples (40 × 40 × 160 mm) have been tested. Table 2 resume all the combinations.

## 3. Analysis of Fresh State Properties

### 3.1. Density

The density of the samples has been measured according to EN-445 [33]. A properly calibrated Gibertini EU-C LCD electronic scale of 0 to 7.5 Kg has been used. The measurements have been taken with the freshly mixed grout. The results show how cases with slag substitution have lower densities than the particular reference case without substitution. It is also noted that mixtures made with Portland 42.5 cement are less dense than those made with Portland 52.5 cement. As expected, in mixtures with a lower water/binder value, density increases. Results are summarized in Table 3, units are gr/cm^3^, and data are the average of data obtained.

### 3.2. Fluidity

This section presents results of the fluidity of the mixtures according to two tests: the Marsh cone and the grout spread test. In both cases, the fresh grout is tested, according to EN 445 [33].

**Figure 3:**
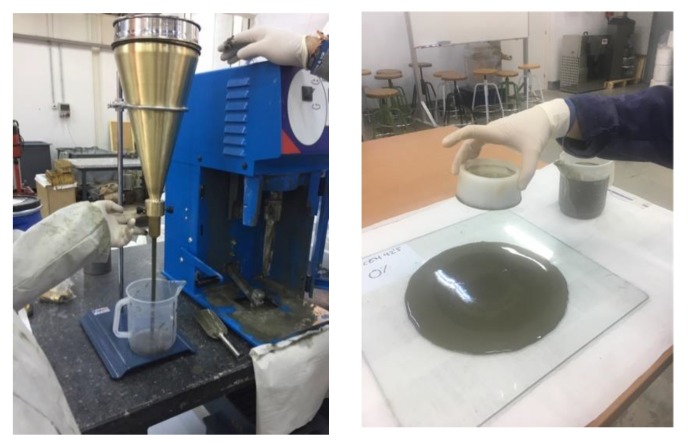
Marsh cone test and grout spread test for fluidity measurements.

Standard EN 447 [17] establishes the requirements applicable to the flow test and the Marsh cone. The test determines the time elapsed until a specific volume of fluid cement grout go through a standardized flow cone. The time to pass through it, immediately after mixing, must be less than 25 s. The grout spread test has been carried out through the use of a small depression cone (70 mm upper diameter, 80 mm lower diameter, and 40 mm height), as indicated in standard UNE EN-445 [33]. The fluidity is measured as a function of the diameter of the circle that forms the grout when spread on a flat plate for a space of 30 s. Examples of both tests are shown in Figure 3.

**Figure 4:**
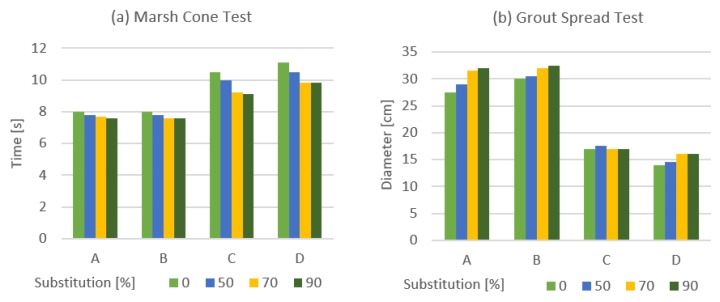
Results of the fluidity test for different mixtures. (**a**) Marsh cone test and (**b**) grout spread test. Consult Table 2 for mixtures identification (**A**, **B**, **C,** and **D**).

Figure 4 presents the results of both fluidity tests. Figure 4a displays Marsh cone data in seconds and Figure 4b shows spread data in centimeters. The fluidity in mixtures with different types of cement but the same water cement ratio behaves very similarly. Although, the percentage of substitution produced a variability of a fluidity property. When the percentage increases, so does the fluidity. This phenomenon stabilizes with substitutions of more than 70%. The variation in fluidity in mixtures C and D is less pronounced.

It is of particular interest to highlight that all mixtures with slag substitution present higher fluidity with respect to mixtures manufactured without slag substitution.

In the spread test, it is observed that mixtures with a one-to-one water/binder ratio (A and B) present higher spreading as we increase slag substitution. However, spreading in the mixtures C and D (w/b ratio 0.67/1) remains practically the same.

### 3.3. Exudation

**Figure 5:**
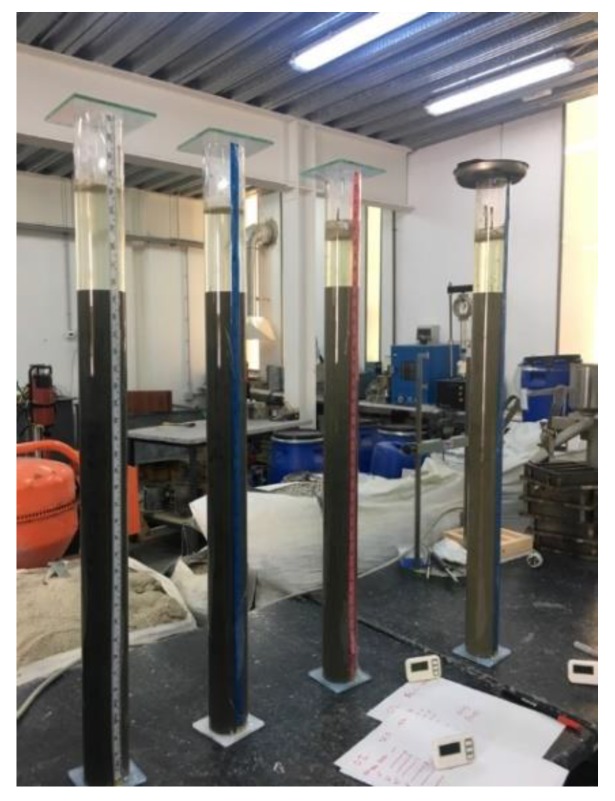
Exudation test.

The exudation test has been carried out in accordance with EN 445 [33]. A transparent tube (65 mm inner diameter and 1 m long) has been used. The tube is oriented in an upright position with the upper end open. It ensures a rigid fixation that prevents any movement or vibration. The grout is poured into the tube with a constant flow to ensure that no additional air is introduced. The tube is filled to the height of ho. The ambient temperature of the laboratory was 18.1 °C and the grout acquires a temperature of 18.3 ° C. The start time t0 and the height h0 are recorded. The height, hg, is recorded at 15-min intervals during the first hour and then at 2-h, 3-h, and 24-h intervals. The height of the exudate water, hw, is recorded at the same time as the grout measurements are made. The possible heterogeneities that can be seen in their appearance through the transparent tube are recorded. The measure of exudation will be the volume of water left in the upper volume of the tube. The volume variation is quantified as the difference in the percentage of the grout volume between the start and the end of the test (Figure 5).

**Figure 6:**
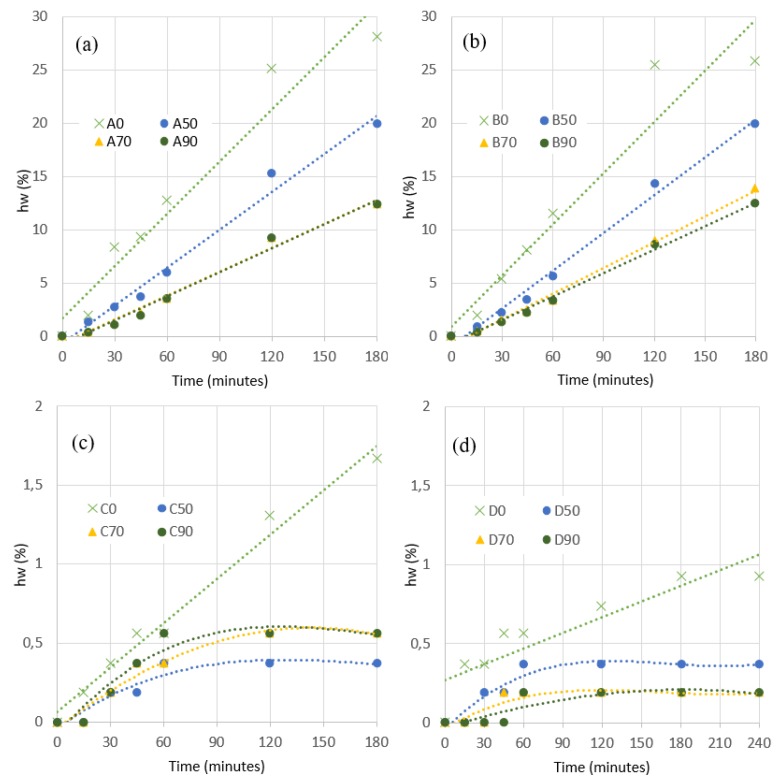
Average results of exudation tests. (**a**) Mixtures A. (**b**) Mixtures B. (**c**) Mixtures C (**d**) Mixtures D.

Figure 6 presents exudation solutions. In all types of mixtures with slag substitution, the exudation decreases with respect to the mixtures without substitution. The higher the % substitution, the lower the exudation. As expected, mixtures with a w/b of 1/1 exude much more than 0.67/1 ratio, in the order of 25%.

## 4. Strength Properties

**Figure 7:**
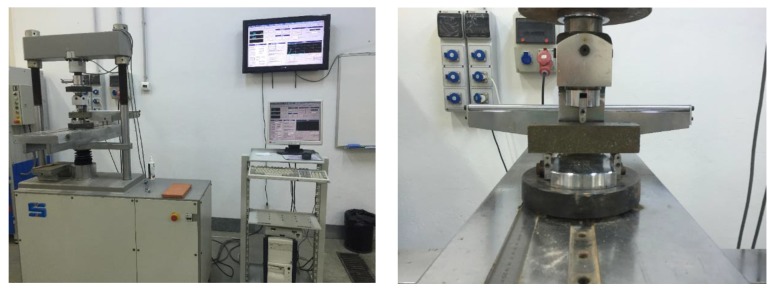
ETIMATIC-Proetisa H0224 used in tests.

The feasibility of using slag instead of cement requires that the resulting mixtures maintain resistant levels suitable for their use. Therefore, one of the main objectives of this study is the mechanical characterization of the specimens. It is necessary to analyze how the percentage of substitution parameter affects the hardening curves. All mixtures have been subjected to standardized tests. The tests were carried out at 2, 4, 7, 14, 28, and 90 days for all the specimens. The two tests used include the flexural strength test and the compressive strength test. Both of them are based on EN 196-1 [32] and EN 196-7 [34] standards for cements. The equipment model was an ETIMATIC-Proetisa H0224 (Figure 7) with an National Accreditation Entity (ENAC) calibration certification.

In the flexural strength test, the force is adjusted by a load cell. The speed was 50 ± 10 N/s. The distance between rollers was 100 ± 0.5 mm. A third roller is situated between them over the specimen (load roller) at a distance of 50 ± 0.5 mm. The strength result is given by the expression below.

(1) $$R_{f}=\frac{1.5\cdot F_{f}\cdot l}{b^{3}}$$

where *R_f_* is the flexural strength (MPa), *F_f_* is the applied load (N), l is the distance between rollers (mm), and b is the square section side (mm).

In a compressive strength test, the compressive strength is given by the expression below.

(2) $$R_{c}=\frac{F_{c}}{1600}$$

where *R_c_* is the compressive strength (MPa), *F_c_* is the breaking load (N), and 1600 is the cross-sectional area (40 × 40 mm).

### 4.1. Analysis of Compressive Strength Evolution

**Figure 8:**
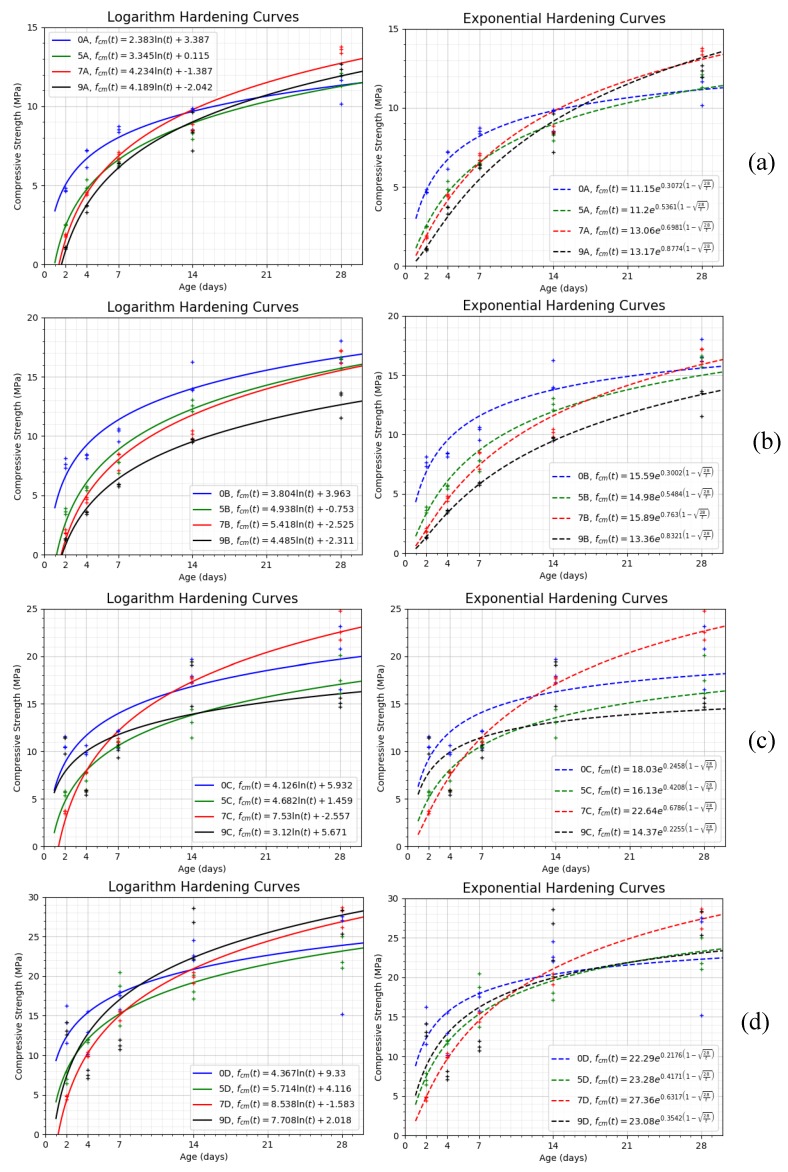
Compressive Strength. Logarithm and exponential hardening curves to 28 days. 0% to 90% substitution. (**a**) A mixtures. (**b**) B mixtures. (**c**) C mixtures. (**d**) D mixtures.

The compressive strength is a key indicator of the feasibility of the mixture with respect to its mechanical response. Each data is the results of six specimens tested. Figure 8 presents the resume of compressive hardening curves obtained. The data curve setting is made on both a logarithmic and an exponential approximation. In the exponential setting, it has been used as the equation include in the Spanish concrete normative (EHE).

(3) $$f_{cm}\left(t\right)=f_{cm,28}\;e^{s\left(1-\sqrt{\frac{28}{t}}\right)}$$

where *f_cm_* is the average compressive strength, *f*_*cm*,28_ is the average compressive strength at 28 days, the *s* coefficient depends on the type of cement, and *t* is the time in days.

Figure 8 presents the results obtained for each combination of cement and water-binder ratio (A, B, C, and D). The reference sample without substitution (A0, B0, C0, and D0) is included. Approaches for the hardening curves, both exponential and logarithmic, of each mixture are provided and their analytical expression are introduced in the figures.

Observing the figures, we can summarize the following.

✓ The existence of slag in the mixture seems to retard the speed of compression hardening. All mixtures present lower compressive strengths in the first days. This delay is also more pronounced the higher the percentage of slags are in the mixture.
✓ In general, the substitution of slag cement maintains the compressive strength of the resulting mixture in the same order of magnitude. Although it presents variations depending on the percentage and the rest of the characteristics of the mixture (type of cement and ratio w/b). In general, all mixtures are appropriate to resist these efforts in uses similar to those of the reference mixture.
✓ Substitutions of 50% from day 14, the results tend to converge to the reference in all cases. This level of substitution offers us a mixture more similar to the original in terms of mechanical capacity independently of the rest of the factors analyzed.
✓ When we increase the percentage, we find an increase in resistance up to 70% in all of them. From there, the behavior of up to 90% shows variations. For type A and D mixtures, 70% and 90% converge. For mixtures type B and C, however, those of 90% worsen their behavior.
✓ We observe cases where we obtain considerable compression strength increases but the time to reach them are variable. A70 presents 70% more resistance. B70 also reach these values but in 90 days (off the chart of the figure). Mixtures C70 and D90 present a lower increase in a range of 35% to 50%.

### 4.2. Analysis of Flexural Strength Results

Table 4 summarizes the average results obtained in the tests for each type of mixture. Data are provided for 2, 4, 7, 14, 28, and 90 days. Each data is the results of three specimens tested. The strength gain obtained in each mixture at 90 days is compared with the reference. The behaviours of the mixtures in this test show similarity with those of the compression test.

✓ In the first days of setting, none of the mixtures with slag substitution achieves the flexural strength of the reference mixture.
✓ Mixtures with 70% substitutions present the best behaviours. From this level, the mixtures tend to stabilize with somewhat lower values (B-D) or bring their resistance closer to that of reference (A-C).
✓ Comparing the mixtures A and B, it can be observed, as expected, that the mixtures follow the same evolution in terms of resistance gained over time. Type B mixtures being made with 52.5 cement reaches more strength.
✓ Comparing mixtures C and D, the evolution is equalized in virtually all mixtures regardless of the type of cement used. The more percentage of substitution used, the more delay in hardening in the first days of setting. In this type of mixture, a resistance gain with respect to the reference mixtures is not reflected up to 90 days.
✓ Comparing mixtures A and C, Type C mixtures reflect better behaviour with respect to the flexural strength gain than type A mixtures.
✓ Comparing mixtures B and D, as in the case of the comparison between mixtures type A and C, the evolution of the flexural strength gains remains unmarked.

## 5. Unsoundness of Slag

**Figure 9:**
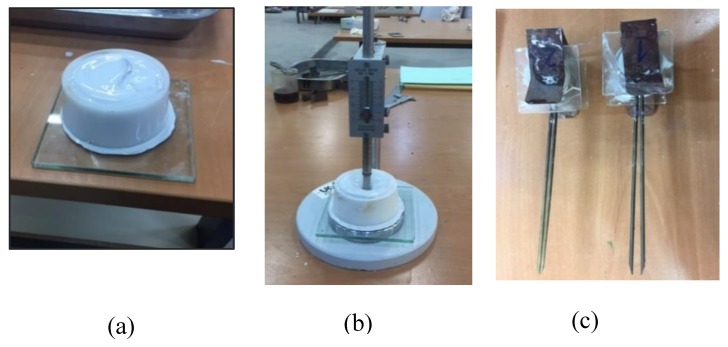
(**a**) GGBS-based paste. (**b**) Consistency. Vicat apparatus. (**c**) Le Chatelier molds.

The expansion of slag has been calculated performing the soundness test. The objective is to compare the soundness of slag versus the cement. This is an important property related to its durability and it is necessary to understand the incidence in the mixture from using slag instead of cement. The Le Chatelier apparatus test has been used in our laboratory, according to the methodology described in the standard EN 196-3 [35]. It has been considered an appropriate procedure for the material used (Figure 9).

The soundness of the slag is the ability to retain its volume after setting in a hardened paste. To obtain the normal consistency, the Vicat apparatus is used. Several pastes were made with different water ratios until one was found that produced a reading of 6 ± 2 mm between the probe and the base plate of Vicat. Once the paste has been introduced into the mold of Le Chatelier, the entire device is placed in the wet chamber. It is maintained for 24 h ± 30 min at (20 ± 1) °C and at a minimum relative humidity of 90%. The mold could also be placed between its plates with the additional mass, if necessary, in a water bath and kept submerged for 24 h ± 30 min at (20 ± 1) °C.

- A distance measurement: At the end of the 24 h ± 30 min period, the distance (A) between the needle tips is measured with an approximation of 0.5 mm. The mold is then gradually heated to boiling for 30 ± 5 min and the bath is kept at the boiling temperature for 3 h ± 5 min.
- B Distance measurement: At the end of the boiling period, the distance (B) between the needle tips can be measured with an approximation of 0.5 mm.
- C Distance measurement: The specimens are removed from the heat, and allowed to cool to the laboratory temperature. The distance (C) between the needle tips is measured with an approximation of 0.5 mm.

Measurements A and C are recorded, and the difference (C-A), to the nearest millimeter, is calculated. If the expansion exceeds the specification limit for the cement reference, a repetition must be carried out. 

Tested pastes of cement and slag were compared in terms of their relative behaviour. The tests’ average results are presented in Table 5. The measurement of distances and the difference between points C and A are expressed in millimeters. The results of the tests carried out show how the slag presents greater soundness than the cement. This implies that it will be able to form a non-disintegrating, harder strong mass on the setting. The conclusion that the slag expands less than the cement is consistent with the exudation test where there is an increase in volume in slag-substituted mixtures.

## 6. Leaching Behaviour and Environmental Influence

In previous sections, we verified how strength characteristics and fresh state properties are similar or superior to those of cementitious mixtures. However, in the analysis of the viability of slag substitution mixtures, it is important to consider additional aspects such as studying their environmental impact.

Therefore, the behavior of slags against cement in the leachate has been analyzed. When solid materials are exposed to a liquid, some of its components can be dissolved and discharged into the environment. The flow by the dissolution of each constituent is of great interest to analyze the level of potential contamination of the material. The leaching of the materials can occur in the place where it is applied by natural infiltration of water, rains, exposure to seawater, etc.

In this work, the grouts with 52.5 cement without slag substitution and the grouts with 70% replacement have been analyzed. The water of exudation for these two cases have also been evaluated.

Table 6 presents the result of leaching expressed as the average value of three instrumental spectroscopy measures. The average values of leaching in mg/L and mg/kg are evaluated when compared with the maximum permissible values of standard EN-14405 [36]. The last column of the table also introduces percolation test results.

It is important to highlight how slag-substituted mixtures show a decrease in the leaching of all chemical components analyzed. It is also observed that, in all elements except chromium (Cr), the values obtained are lower than those of the standard. This was expected since the standard establishes different conditions for data collection. However, the important point is that the slag mixture has a value of 0.853 mg/L (Cr) compared to 1.23 mg/L (Cr) of the reference mixture. Therefore, we can conclude that the substitution of cement with slag in the mixture decreases the leachate and produces a mixture with less environmental impact. It is considered of special interest to evaluate the percolation water that can be filtered to the ground. As can be seen in the results’ table, the leaching of all components in mixtures with substitution of slags is also less than in those made only with cement.

## 7. Conclusions

In this work, a comprehensive set of tests has been carried out to analyze the behavior of slurries with slag replacement. Attention has been given to all mixtures running without additives and under the same conditions in our laboratories. In addition to the usual tests of mechanical resistance and fresh properties, special interest has been placed in studying responses related to their impact on the environment. We highlight the results obtained in terms of expansiveness and leaching of the mixtures. We summarize the main conclusions that emerge from the results and the discussions described in previous sections.

- Mixtures with slags are less dense than mixtures without substitution, which becomes more fluid as it increases the percentage substitution.
- In all types of mixtures, the exudation decreases with respect to the mixtures without substitution. The higher the percentage, the lower the exudation. As expected, mixtures with 1/1 water/binder ratio exudes much more than 0.67/1 ones in the order of 25%.
- Results prove a significance reduction in chemical element leaching in all the mixtures with slag substitution. The expansion is lower as well.
- The mechanical response in terms of flexion and compression strength gives better results in all types of mixtures with substitution. The hardening process of the mixture is modified and delayed. At an early stage, the resistance of the reference mixture is not reached. However, the hardening period increases and the final resistances after 90 days are obtained. Optimal mixtures are those made with the percentage of 70% substitution.
- The slower development of mechanical properties in an early age have to be considered in potential applications of these mixtures.
- Mixtures with a high percentage of substitution (90%) present an interesting behavior related to strength properties. Both cases, with CEM I 52.5R and CEM I 42.5 R cement, produce similar levels of resistance. In these study cases, the cement loses relevance with respect to slag used in terms of its mechanical characterization.
- It is also observed that the mixtures made with slag substitution and with water/binder 1/1 are the ones that offer the greatest increase of strength due to the greater hydration.
- High substitution of cement with GGBFS may lead to the problem of volume stability. It has to be properly cured.

## Figures and Tables

**Table 1:** Chemical composition of cement and GGBFS.

Oxides	CEM I 42.5 and 52.5 Composition (%)	GGBFS Composition (%)
CaO	63.56	47.14
SiO_2_	19.30	32.30
SO_3_	2.91	1.52
Al_2_O_3_	5.57	8.90
Fe_2_O_3_	3.46	0.29
MgO	0.86	7.64
K_2_O	0.80	0.45
Na_2_O	0.13	0.08
Other (ZnO, MnO, ZrO_2_, …)	3.41	1.97

**Table 2:** Resume of mixtures identification.

Series	Id	Mix Composition		
		w/b	Cement	GGBFS (%)
A	A0	1/1	42.5	0
	A50	1/1	42.5	50
	A70	1/1	42.5	70
	A90	1/1	42.5	90
B	B0	1/1	52.5R	0
	B50	1/1	52.5R	50
	B70	1/1	52.5R	70
	B90	1/1	52.5R	90
C	C0	0.67/1	42.5	0
	C50	0.67/1	42.5	50
	C70	0.67/1	42.5	70
	C90	0.67/1	42.5	90
D	D0	0.67/1	52.5R	0
	D50	0.67/1	52.5R	50
	D70	0.67/1	52.5R	70
	D90	0.67/1	52.5R	90

**Table 3:** Fresh density results (gr/cm^3^).

% Mix ID	A	B	C	D
**0**	1.47	1.44	1.82	1.77
**50**	1.44	1.45	1.79	1.75
**70**	1.46	1.40	1.77	1.74
**90**	1.42	1.40	1.76	1.72

**Table 4:** Flexural strength results (MPa).

	Days	2	4	7	14	28	90	Increase with Respect to Reference
Mixture								
0A		1.61	2.11	2.45	2.86	3.35	3.92	-
50A		0.94	1.39	1.65	2.41	3.46	4.63	18.11%
70A		0.69	1.57	2.22	2.55	3.34	4.72	20.41%
90A		0.44	0.98	1.44	2.03	2.94	4.05	3.32%
0B		2.68	3.14	3.51	4.78	5.21	5.46	-
50B		0.96	1.96	2.94	3.89	4.35	5.98	9.52%
70B		0.78	1.75	2.67	4.01	4.96	6.2	13.55%
90B		0.51	1.36	2.16	3.55	3.28	6.12	12.09%
0C		3.36	3.54	3.92	5.3	6.23	6.54	-
50C		1.66	2.77	3.51	4.58	5.38	6.8	3.98%
70C		1.2	2.08	3.3	4.21	5.69	7.45	13.91%
90C		0.46	1.75	2.03	4.44	4.77	6.76	3.36%
0D		4.55	5.13	5.4	5.67	6.09	7.03	-
50D		2.38	3.84	4.24	4.76	5.69	7.49	6.54%
70D		1.66	3.14	3.23	5.06	6.4	8.02	14.08%
90D		0.61	1.4	1.95	4.43	6.03	7.63	8.53%

**Table 5:** Le Chatelier soundness test results.

	Cement-Based Paste				GGBS-Based Paste			
	A (mm)	B (mm)	C (mm)	C-A (mm)	A (mm)	B (mm)	C (mm)	C-A (mm)
Specimen 1	7	8.2	9	2	7	7.5	7.5	0.5
Specimen 2	10	12.4	13	3	8	8.5	8.5	0.5

**Table 6:** Leaching results of B0 and B70 mixtures.

Components	Water Exudation from B0 (mg/L)	Water Exudation from B70 (mg/L)	B0 Mix (mg/L)	B0 Mix (mg/kg)	B70 Mix (mg/L)	B70 Mix (mg/kg)	Reference from EN-14405 (mg/kg)	Percolation mg/L
weight (kg)			0.35	0.35	0.35	0.35		
[Na] mg/L	680	336	11.6	33.1429	4.51	12.8857	-	-
[K] mg/L	5780	2872	47.8	136.5714	20.2	57.7143	-	-
[Ca] mg/L	571	805	185	528.5714	4.73	13.5143	-	-
[Mg]mg/L	0.014	0.021	0.0202	0.0577	0.076	0.2171	-	-
[Al] mg/L	0.001	0.0029	0.17	0.4857	0.703	2.0086	-	-
[Si]mg/L	0.013	0.011	0.0086	0.0246	0.044	0.1257	-	-
[Ti] mg/L	< 0.0001	< 0.0001	< 0.0001	-	< 0.0001	-	-	-
[Cr] mg/L	1.23	0.853	0.015	0.0429	0.00016	0.0005	0.5	0.1
[Mn] mg/L	0.0001	0.0003	0.0001	0.0003	0.00068	0.0019	-	-
[Fe] mg/L	0.001	0.0023	0.0182	0.0520	0.006	0.0171	-	-
[Ni] mg/L	0.00029	0.00017	0.00027	0.0008	0.000174	0.0005	0.4	0.12
[Cu] mg/L	0.00413	0.0031	0.00192	0.0055	0.0045	0.0129	2	0.6
[Zn] mg/L	0.0021	0.00089	0.0066	0.0189	0.000694	0.0020	4	1.2
[As] mg/L	0.000541	0.00039	< 0.0002	-	0.00033	0.0009	2	0.06
[Cd] mg/L	< 0.0001	< 0.0001	< 0.0001	-	< 0.0001	-	1	0.02
[Sn] mg/L	< 0.0001	< 0.0001	< 0.0001	-	< 0.0001	-	-	-
[Pb] mg/L	0.0082	0.002	0.000543	0.0016	< 0.0001	-	0.5	0.15
